# Identification of differentially expressed sense and antisense transcript pairs in breast epithelial tissues

**DOI:** 10.1186/1471-2164-10-324

**Published:** 2009-07-17

**Authors:** Anita Grigoriadis, Gavin R Oliver, Austin Tanney, Howard Kendrick, Matt J Smalley, Parmjit Jat, A Munro Neville

**Affiliations:** 1 Ludwig Institute for Cancer Research, 605 Third Avenue, New York, NY 10158, USA; 2 Almac Diagnostics, 19 Seagoe Industrial Estate, Craigavon, Northern Ireland, BT63 5QD, UK; 3 The Breakthrough Breast Cancer Research Centre, The Institute of Cancer Research, 237 Fulham Road, London, SW3 6JB, UK; 4 Department of Neurodegenerative Disease, Institute of Neurology, London, WC1N 3BG, UK; 5 Breakthrough Breast Cancer Research Unit, Guy's Hospital, King's Health Partners AHSC, London, UK

## Abstract

**Background:**

More than 20% of human transcripts have naturally occurring antisense products (or natural antisense transcripts – NATs), some of which may play a key role in a range of human diseases. To date, several databases of *in silico *defined human sense-antisense (SAS) pairs have appeared, however no study has focused on differential expression of SAS pairs in breast tissue. We therefore investigated the expression levels of sense and antisense transcripts in normal and malignant human breast epithelia using the Affymetrix HG-U133 Plus 2.0 and Almac Diagnostics Breast Cancer DSA microarray technologies as well as massively parallel signature sequencing (MPSS) data.

**Results:**

The expression of more than 2500 antisense transcripts were detected in normal breast duct luminal cells and in primary breast tumors substantially enriched for their epithelial cell content by DSA microarray. Expression of 431 NATs were confirmed by either of the other two technologies. A corresponding sense transcript could be identified on DSA for 257 antisense transcripts. Of these SAS pairs, 163 have not been previously reported. A positive correlation of differential expression between normal and malignant breast samples was observed for most SAS pairs. Orientation specific RT-QPCR of selected SAS pairs validated their expression in several breast cancer cell lines and solid breast tumours.

**Conclusion:**

Disease-focused and antisense enriched microarray platforms (such as Breast Cancer DSA) confirm the assumption that antisense transcription in the human breast is more prevalent than previously anticipated. Expression of a proportion of these NATs has already been confirmed by other technologies while the true existence of the remaining ones has to be validated. Nevertheless, future studies will reveal whether the relative abundances of antisense and sense transcripts have regulatory influences on the translation of these mRNAs.

## Background

Naturally occurring antisense transcripts (NATs) are sequences complementary to other transcripts and were first identified in prokaryotes and viruses, where their expression influences mRNA transcription, processing and translation [1]. Over the past few years, antisense transcription in human and other eukaryotic genomes has become increasingly evident due to the availability of high throughput sequencing technologies and strand-specific tiling oligonucleotide arrays [2-4]. The rigorous analysis of the human genome by the ENCyclopedia Of DNA Elements (ENCODE) project fortified the notion that transcription is substantially more complex than previously conceived and that at least 15% of all transcripts could derive from antisense transcription [5]. So far more than 10,000 potential sense/antisense (SAS) transcript pairs have been identified in both human and mouse genomes [6] and several antisense containing databases such as antiCODE have been published [7]. While the majority of studies have focused on the mapping and evolutionary aspect of SAS pairs, only a few studies have interrogated and validated their abundance in different human tissues [2,6,8-10]. Here we report for the first time a comprehensive analysis of SAS pairs with regards to their differential expression in the normal and malignant breast epithelium.

A discrete function of SAS pairs in human tissues has not been identified, although their regulatory activity on protein expression at diverse levels, such as alternative splicing, post-transcriptional regulation, transport and epigenetic imprinting as well as transcriptional and translational interference through annealing to complementary sequences has been postulated [11]. Since some functional data for their involvement in developmental processes have recently emerged [12,13], their role in malignant transformation of human tissues may be foreseen.

Initially, NATs were identified using large collections of mRNA, genomic and EST sequences, as well as expression data from methods such as serial analysis of gene expression (SAGE) and massively parallel signature sequencing (MPSS) [6,14-16]. By using these sequencing data for the identification of NATs, stringent criteria were applied to determine correctly the orientation of each sense or antisense transcript relative to its genomic sequence. Over the last few years microarray based experiments have validated the prevalence of NATs in different human and mouse tissues [4,5,8,10]. Using strand-specific microarray probes, Oeder *et al*. interrogated the abundance of many different NATs based on the mouse Affymetrix MOE430 microarray dataset [8], while Ye *et al*. identified several hundred NATs in 19 human cell lines [2]. Ge and colleagues extended the use of oligonucleotide based microarrays for the analysis of NAT expression by altering the protocol for cDNA synthesis and thereby strongly supporting the observation that encoding of transcripts on both DNA strands often results in complementary mRNAs [17]. Recently, another human microarray chip based on the Affymetrix Genechip platform – namely the Breast Cancer DSA – was developed by Almac Diagnostics [18]. The design methodology of the DSA range of research tools has previously been described [19]. In brief, the Breast Cancer DSA microarray was designed utilising breast cancer-specific sequence information consisting of full length public mRNAs and contigs generated by the assembly of a range of public and proprietary EST datasets. The DSA is classified as a discovery platform and its content ranges from well-characterised transcripts to those whose function is currently unknown. Furthermore, the content includes a large number of probe sets specific for the detection of antisense transcripts in addition to a significant amount of sense transcript information not found on other commercially available microarrays. It is therefore ideally suited to investigate the sense and antisense transcriptome of breast cancer.

In this study, we have used the Breast Cancer DSA research tool in conjunction with the Affymetrix HG-U133 Plus 2.0 GeneChip and MPSS data sets of our established differential tumour epithelial transcriptome [20] to analyze the expression of SAS pairs in immunomagnetically separated normal human luminal epithelial cells and primary breast cancers substantially enriched for their neoplastic epithelial component. The aim of the current study was not only to identify SAS pairs expressed in the epithelial cells of human breast tissue, but also to validate their expression signature using several different technologies. Our particular focus was upon SAS pairs with deregulated expression in mammary epithelial cells, and to ascertain whether the expression of the antisense transcripts could be detected in several different breast cell lines and solid primary breast tumours. Generating the first comprehensive dataset of SAS pairs in epithelial breast tissue, our analysis has also shed light on the nuances of antisense and sense transcription.

## Results

### Microarray probe sets and MPSS tags with sense and antisense orientation

The basis of all cross-platform comparison is reliability of annotation and mapping of microarray features and tag sequences, especially if transcripts from the sense or antisense orientation are to be distinguished. To ensure the correct annotation, probesets from both microarray platforms, as well as MPSS tags, were mapped by sequence alignment to a human transcriptome (HTR) database that was previously used for a multiple platform comparison study [20,21]. To be included for further analysis, stringent filtering criteria were applied to the probe sets and MPSS tags: firstly, probe sets and MPSS tags that could not be mapped to a HTR cluster or which aligned *in silico *to several clusters were eliminated; secondly, probe sets had to exclusively detect either the sense or antisense transcript – which was determined by the orientation of alignment of the probes or tags to the HTR clusters.

Both microarrays had approximately the same number of microarray features printed, of which 33,355 out of 60,854 (54%) and 38,047 out of 54,613 (69%) probe sets were unambiguously mapped to a HTR cluster in sense orientation, for the Breast Cancer DSA and Plus 2.0, respectively (Table 1). In contrast, 8,426 out of 60,854 (14%) probe sets on Breast Cancer DSA could potentially detect antisense transcripts which is more than double than the number detected by the Plus 2.0 (3,476 out of 54,613 or ≈ 6%) (Table 1). Despite the fact that the overall magnitude of transcript detection by MPSS was much smaller than those of the two microarray platforms, the percentage of tags mapping to the sense strand to the overall detected sequences was similar to the microarray platforms (69%; 13,611/19,794). Due to the MPSS technology and our stringent criteria for inclusion as a potential antisense detectable tag, only 215/19,794 (1%) MPSS tags could potentially be derived from antisense transcripts (Table 1).

**Table 1:** Sense and antisense mapping of probe sets and sequence tags

	***SENSE ORIENTATION***			***ANTISENSE ORIENTATION***		
	*Plus 2.0*	*DSA*	*MPSS*	*Plus 2.0*	*DSA*	*MPSS*
*Plus 2.0*	21,078 HTR			2,995 HTR		
*DSA*	16,549 HTR	17,737 HTR		1,753 HTR	6,358 HTR	
*MPSS*	7,856 HTR	7,878 HTR	8,452 HTR	51 HTR	101 HTR	203 HTR

Numbers of representative human transcriptome clusters (HTR) Mapping of the *Affymetrix HG U133 Plus 2.0 GeneChip *(Plus 2.0) probe sets, *Almac Diagnostics Breast Cancer DSA *(DSA) probe sets and *massively parallel signature sequencing *(MPSS) sequence tags to the human transcriptome clusters (HTR) in either sense or antisense orientation.

Based on the HTR database, transcript coverage of sense and antisense probes of these three technologies was compared. Since both the Affymetrix probesets and the HTR database were based solely on public sequence data, the Plus 2.0 had the highest coverage of sense-mapped features. 16,549 (78%) of those HTR clusters were also represented on Breast Cancer DSA, as were an additional 1,287 clusters not detected on the Plus 2.0 (Table 1). The limitation of the MPSS sequencing technology became apparent in this comparison, identifying only a third of all sense transcripts represented on these microarray chips (7,856 HTR common with Plus 2.0; and 7, 878 HTR clusters with Breast Cancer DSA). Interestingly, when the coverage of antisense transcripts was compared between these three technologies, a different picture was obtained. The majority of antisense-containing HTR clusters was found on the Breast Cancer DSA. Out of the 6,358 antisense-containing HTR clusters 1,753 overlapped with the Plus 2.0 and 101 HTR clusters were also identified by MPSS, illustrating the enrichment of antisense transcripts on the Breast Cancer DSA microarray platform. The full HTR mapping information for the DSA microarray features [see Additional file 1], Plus 2 microarray features [see Additional file 2], and MPSS tags [see Additional file 3] are provided, and may provide a useful resource not only for inclusion in current antisense databases, but also to interrogate possible antisense transcription in several human tissues and in published human expression datasets. HTR database sequences are available on request.

To determine which of these antisense strand-matching microarray features showed expression in the human breast epithelium, the absence or presence calls for all probe sets on both microarray platforms in either the normal luminal epithelial or the malignant breast epithelial sample were established using the MAS5 algorithm. Microarray features were included for further studies if at least two out of three technical replicates per platform agreed in their present calls, while MPSS tags were kept when their tag count was at least 3 tags per million. Their corresponding HTR cluster identifier was used to represent MPSS tags and microarray features. It was noted that more than 60% of all probe sets passed the present call criteria on the Breast Cancer DSA, only 39% had a concordant present call on the Plus 2.0. As a next step, the sense and the antisense transcriptomes of the normal and the malignant breast epithelia were compared. Table 2. shows an initial comparison of present calls obtained using the two microarray platforms and MPSS in both normal and malignant cell-lines. These numbers provide an initial view of the number of transcripts detectable by each of the three technologies.

**Table 2:** HTR mapped probesets and tags called present.

	**Total number of probesets mapped to HTR**	**Total mapped probesets present in *sense *normal breast epithelium**	**Total mapped probesets present in *antisense *normal breast epithelium**	**Total mapped probesets present in *sense *malignant breast epithelium**	**Total mapped probesets present in *antisense *malignant breast epithelium**
***DSA***	41,781 (100%)	12,330 (29%)	2,407 (5.8%)	13,408 (33.3%)	2,728 (6.5%)
***Plus 2.0***	41,523 (100%)	9,655 (23.2%)	433 (1%)	10,447 (25.2%)	131 (0.3%)
***MPSS***	13,826 (100%)	5,614 (40%)	94 (0.7%)	7,329 (53%)	155 (1.1%)

Total number of probesets/tags mapped to the HTR database and the number of present calls seen in normal and malignant breast epithelium in both sense and antisense orientations. These numbers indicate the actual detections levels of each technology.

**Figure 1:**
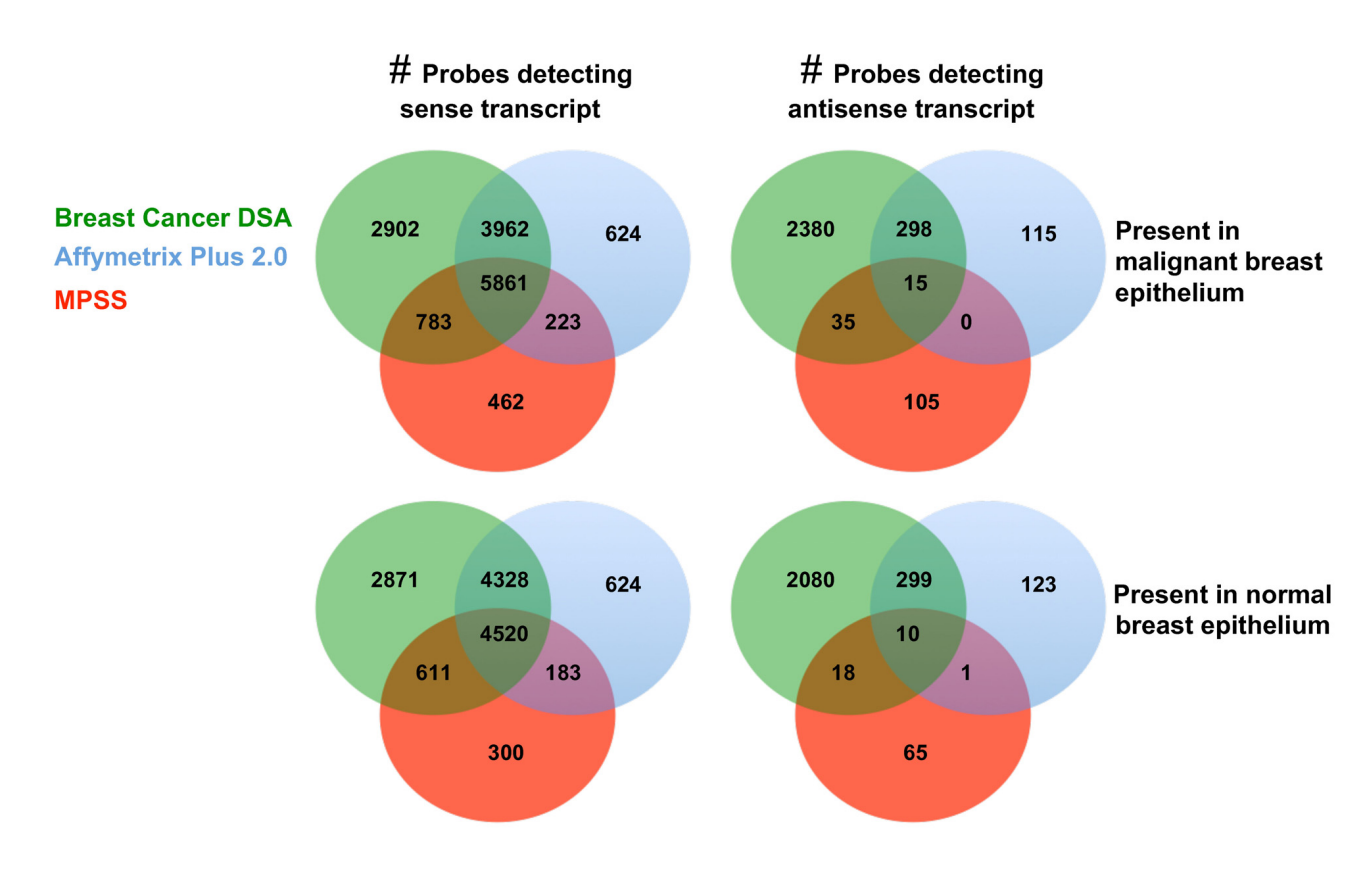
**Overlay of expression detection between MPSS and microarray for sense and antisense transcripts malignant and normal breast epithelium**. Human transcriptome clusters (HTR) were used to measure the concordance in detecting sense and antisense transcripts for the Breast Cancer DSA (green), Affymetrix HG U133 Plus 2.0 (blue) and MPSS (red) in both the malignant breast epithelium and normal luminal epithelium. The presence of a MPPS tag was determined if a MPSS tag had at least a count of 3 tpm in these samples. For the two microarray platforms, a HTR cluster was called present when its containing sense (top section) or antisense (bottom section) probe set obtained a present calls with the MAS5 algorithm.

As shown in Figure 1, the two microarray platforms had a concordance of ~9,000 transcripts in the normal setting, but nearly five times more transcripts were detected specifically on the breast tissue specific Breast Cancer DSA microarray platform in the normal and malignant epithelium (Figure 1). Furthermore, expression profiles obtained by the Breast Cancer DSA identified three times more transcripts found by MPSS than the Affymetrix platform. This difference between the generic Affymetrix Plus 2.0 and the tissue-specific Breast Cancer DSA became even more apparent when antisense-containing HTR clusters were compared. While 43% of all antisense-detecting probes sets obtained a present call, only 14% on the Plus 2.0 could be detected, corresponding to the unique detection of > 2,000 antisense-containing HTR clusters in the normal and the malignant breast epithelium by Breast Cancer DSA. For the DSA platform, 2452 antisense detecting probe sets were in common between the normal and the malignant epithelium, of which 868 showed more than two-fold difference in expression level with a pValue < 0.05. In contrast, 344 Affymetrix probe sets mapping to antisense transcripts were detectable in normal and malignant breast epithelium, of which 60 passed the same criteria, and only 48 MPSS tags showed different expression level.

Selecting those HTR clusters that mapped solely in antisense orientation and were detected by at least 2 of the 3 technologies created a set of 431 'robust' antisense sequences. Sequences could be detected in both the normal and the malignant setting or in either setting individually [see Additional file 4]. As shown in Figure 1, the majority of these commonly represented antisense HTR clusters were represented on the Breast Cancer DSA with the exception of one, which was only detected by the Plus 2.0 and MPSS in the normal luminal breast sample.

### Novel sense-antisense pairs in the human breast tissues

**Figure 2:**
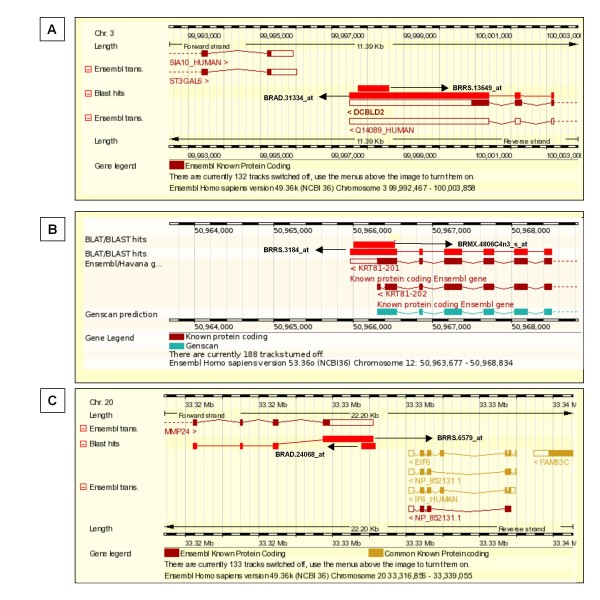
**Alignment of SAS pairs on genome**. Exemplary screenshot of the contig view panel from the ENSEMBL Genome Browser for DCBLD2 (A), KRT81 (B) and MMP24 (C) illustrating probesets overlapping in sense and antisense orientation. SAS pairs are shown as red blocks, aligning to the genome (chequered bars) and known or predicted genes. A leftward arrow denotes alignment to the reverse strand of the genome whilst a rightward arrow represents alignment to the forward strand. Arrows are labeled with the DSA probeset ID representative of the transcript.

Having established a robust set of antisense containing HTR clusters encompassing 431 antisense transcripts, we interrogated how many of those had a corresponding sense transcript also represented on the Breast Cancer DSA. Sense and antisense transcript pairs were defined when their sequence overlapped on opposite strands of the HTR database (see Figure 2). According to our criteria, 257 SAS pairs were identified. Galante *et al*. published a comprehensive study on antisense sense transcripts derived from publicly available sequencing data [6]. To establish if our SAS pairs had been reported previously, their sequences were analysed against the Galante database. Interestingly, only 94 of the 257 SAS pairs produced matches against the database, while the remaining 163 produced no significant alignments and can therefore be considered as novel or previously unidentified. Probeset mapping for the 163 HTRs is provided [see Additional file 5].

**Figure 3:**
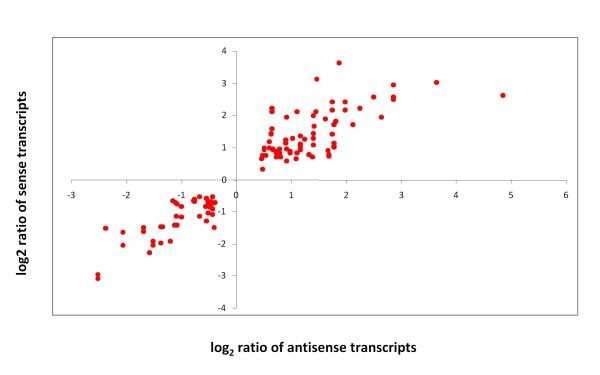
**Correlation of differential gene expression for SAS pairs**. The differential gene expression between the normal and the malignant breast epithelium was determined for all sense and antisense transcripts of the SAS pairs. Using Pearson's correlation, the log2 expression ratios of the sense transcripts were compared with the log2 expression ratios of the antisense transcripts for each SAS pair.

As a next step, we wanted to interrogate if the antisense and sense transcripts of the SAS pairs showed similar expression patterns in the breast epithelium. The differential expression between the normal and the malignant breast epithelium for all SAS pairs was established and only probe sets whose differential expression had a significance level below 0.05 were used. When the Pearson's correlation coefficients for the log2 ratios of our SAS pairs were calculated, all SAS pairs showed a positive correlation as shown in Figure 3.

### Confirmation of differentially expressed SAS pairs by strand-specific qPCR

**Figure 4:**
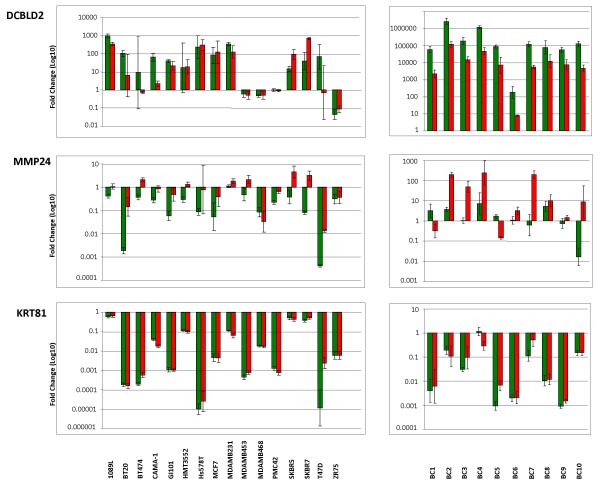
**Quantitative strand-specific RT-PCR analysis of SAS pair expression in breast cancer cell lines and solid primary breast tumours**. cDNAs of 15 breast cancer cell lines and 10 primary breast tumours (BC) were analyzed using the ΔΔCT relative quantification real-time qPCR. Red bars represent sense transcripts, green bars the corresponding antisense transcripts. Analysis of qPCR data was performed using the immortalised luminal cell line (226L) as comparator for all the breast cell lines and solid primary breast tumours (indicates as BC). ACTB was used as endogenous control throughout all analysis. ΔΔCT relative quantification data are expressed as mean fold changes across samples together with 95% confidence intervals.

Since none of the identified SAS pairs exhibited an opposite fold change between the normal and the malignant breast epithelium, we wanted to explore further whether these SAS pairs were exclusively showing positive differential expression patterns. Three of the 163 novel SAS pairs were randomly selected and strand-specific RT-PCR used to measure their expression. The 3 pairs, corresponding to discoidin, CUB and LCCL domain containing 2 (DCBLD2, NM_080927.3), matrix metallopeptidase 24 (MMP24, NM_006690.3) and keratin 81 (KRT81, NM_002281.3) had their expression interrogated in 16 breast cancer cell lines and ten solid primary breast tumours (Figure 4). To distinguish expression coming from the sense or the antisense strand, the first strand cDNA synthesis of the RT-PCR was set up either with the sense primer (generating cDNA from sense strand mRNA transcripts) or the antisense primer (generating cDNA from RNA from the antisense strand). FAM labeled fluorescent probes for each gene were used to determine the relative expression levels of the sense and antisense by qPCR. KRT81 showed similar expression patterns in all tested samples with regards to sense and antisense transcription and overall expression levels. In contrast, DCBLD2 and MMP24 showed significantly higher expression ratios in the solid tumours in comparison with the breast cancer cell lines. Furthermore the sense and antisense transcripts of DCBLD2 had different expression levels in BT474 and T47D, two hormone-receptor positive luminal-specific breast cancer cell lines. When the expression of the antisense and sense MMP24 transcript was interrogated, opposite expression levels were observed in five breast cancer cell lines (namely MDA-MB-231 and HMT3552 of basal-subtype; and BT474, MDA-MB-453, SKBR5 and SKBR7 of luminal-subtype) as well as in five solid breast tumours.

## Discussion

The initial goal of our study was to perform comparative expression profiling of normal and malignant breast epithelia using three different approaches – two commonly used technologies we had utilised previously [21] and a recently released microarray designed specifically for investigation of breast cancer.

To ensure fair comparison, a human transcriptome database was used as a common mapping point for the three technologies [22]. This produced similar numbers of mapped features for the Plus 2.0 and Breast Cancer DSA and markedly lower amounts for MPSS. The similar overall numbers of Plus 2.0 and DSA probesets unambiguously mapping to the HTR database were contrasted when the orientation of alignment was considered, with the Plus 2.0 showing slightly higher numbers of sense features and the DSA showing significantly higher numbers of antisense transcripts. This is most likely explained by the Plus 2.0 design being focused on common protein coding genes from older public data while the DSA design employed tissue specific sequencing and is more likely to have discovered novel content. Therefore whilst the Plus 2.0 could be expected to have more features mapping in the sense orientation, a proportion of these likely represent transcripts not necessarily of functional importance in breast cancer.

The fact that MPSS produced fewer unambiguous maps than either of the other two technologies is a reflection of the short tag length utilised by the technology and the consequent increased likelihood of cross hybridisation. This fact highlights a major shortcoming of the MPSS approach. Whereas either of the microarray technologies utilise probesets containing eleven 25-mer probes and specially formulated to be reflective of a single transcript, the MPSS approach generates single tags only 21 nucleotides in length. This fact creates significant potential for incorrect mapping of tags and means that much of the initial data generated becomes unusable when a stringent mapping methodology is utilised. This serves as a general reminder of the care that must be taken in the interpretation of any data generated on the basis of short, single tags or probes.

Detection analysis showed further advantages of the DSA approach with higher numbers of present calls than the Plus 2.0 array in both orientations. The fact that this trend was observed regardless of the Plus 2.0's higher number of sense mapped features is again suggestive of the advantage of the disease-focused approach used in the generation of the DSA – it would appear that a larger proportion of the Plus 2.0 content does not show expression in breast epithelia. MPSS again under performed at this point, which is reflective of previous assessments of the technology [23].

Generic arrays have previously been suggested as a viable means of studying antisense transcription [8,24] however the higher number of antisense transcripts and higher detection levels on the DSA suggest that antisense transcription would be better studied using a focused approach like the Breast Cancer DSA research tool. Furthermore, the DSA achieved greater concordance with MPSS data than the Plus 2.0 which is noteworthy as our previous studies conducted in the absence of the DSA had identified the Affymetrix Plus 2.0 as the microarray platform that had the highest concordance with the MPSS data set [20].

Our criteria for selection of a 'robust' set of antisense transcripts meant that a large proportion (~90%) of the DSA's antisense probesets were excluded from further analysis. It is likely that some of these antisense transcripts arose due to experimental artefacts [21] and the use of actinomycin D during reverse transcription could have reduced the number of antisense transcripts as seen in the study of Perocchi et al [25]. Nevertheless, it is equally possible that many of these are probesets to genuine antisense sequences and could have yielded useful data – 868 probe sets on the DSA showed more than two fold differential expression, however in the absence of an extended validation of the antisense transcripts it was felt that they should only be considered when confirmed by one of the other two technologies used in the study. This leaves a substantial subset of remaining antisense transcripts whose expression in the breast tissue has to be validated by different technologies in the future.

The 257 robust sense-antisense pairs investigated on the DSA showed a high degree of novelty when compared to a recently created SAS database, suggestive of the fact that a large number of SAS pairs remain to be discovered and reported. Numerous SAS databases have been published by other researchers [7,26] and comparison with these could form the basis of further studies. The large number of novel SAS pairs identified here is understandable as the discovery of antisense transcripts and SAS pairs is still considered a relatively new phenomenon in many quarters and work in this area has yet to reach maturity. This provides further indication of the potential value of the antisense transcripts represented on the DSA but excluded from this study. The nature and function of the 431 'robust' antisense candidates and the subset of these forming the 257 SAS pairs is currently unknown. As stated previously, the Breast Cancer DSA is a discovery platform containing many transcripts that have not yet been well characterised. Whilst we have demonstrated the expression of these antisense transcripts, extensive subsequent validation would be required to elucidate their function and falls outside the scope of the current study. Sequence alignment data for the SAS pairs are provided [see Additional file 5] and may prove a useful resource for future functional analysis.

SAS pairs have previously been classified as head-to-head, tail-to-tail or embedded based on their pattern of overlap [6]. A limitation of the DSA technology is that it utilises 3' biased protocols and therefore only the 3' end of transcripts are interrogated. As a result, SAS pairs discovered using this technology will solely represent tail-to-tail overlap patterns. This fact also suggests that there may be a large body of alternatively classified SAS pairs to be discovered by other experimental means.

The fact that all SAS pairs differentially expressed between the normal and malignant settings showed positive correlation was surprising as negative correlation has previously been reported in several studies [11]. This led us to attempt validation of the SAS expression in a range of malignant cell-lines and solid tumours by means of strand-specific RT-PCR. The results produced by this approach largely correlated with those obtained on the DSA platform, however negative correlation was observed in 13 of the 81 tested samples. So while our pooled samples suggested positive correlation of differential expression of all SAS pairs between normal and malignant settings, individual assessment of a range of solid tumours and cell-lines indicated the existence of alternative patterns of differential expression. While differential expression of the sense and antisense transcript for MMP24 was more prominent in luminal breast cancer cell lines (3/5), significant different expression levels of the DCBLD2 -SAS pair were observed solely in two luminal, hormone receptor positive breast cancer cell lines. This data might suggest that the level of expression for certain SAS pairs could be breast cancer subtype specific. Nonetheless our studies suggest that coexpression of SAS pairs may be more prevalent than inverse expression. The differing patterns of differential expression between samples suggests a potential functional relevance of sense-antisense expression patterns as has previously been reported [11] and serves to highlight the importance of SAS profiling in cancer research. Such knowledge could be beneficial in the elucidation of pathways in cancer and might be exploited in potential future treatments like antisense therapy [27]. Aberrations in SAS expression patterns might well be indicative of disease or could prove useful in sub-classification of a given disease, potentially aiding in the development of targeted treatments.

## Conclusion

In conclusion, the data presented in this study demonstrate a clear benefit in the use of a disease-focused platform such as the Breast Cancer DSA research tool for disease-specific studies. Utilising only a subset of the SAS pair data available on the platform we have shown expression of several hundred SAS pairs, of which a large proportion appear to be novel. We have also identified the expression of many more antisense transcripts not identified by other means. These findings would suggest that many more SAS pairs remain to be discovered and deposited in public databases. RT-PCR has validated the expression of a selection of these SAS pairs and identified patterns of SAS expression that support previous findings and appear to suggest functional relevance. While much of the work presented here is preliminary it still provides a strong indication of the importance of SAS expression in breast and other cancers and highlights that much investigation is still required in this field of research.

## Methods

### Biological samples

RNA samples were isolated from two sources: one pool of 10 primary cultures (~10^7^) of normal human breast luminal cells which were prepared from reduction mammoplasty samples by double immunomagnetic sorting methods [28-30], and one pool of 16 primary breast tumours substantially enriched for epithelial cells immunomagentically purified using FAP antibody. RNA purity and integrity was assessed with an Agilent 2100 Bioanalyzer (Agilent Technologies, Palo Alto, CA). Cell purification procedures as well as details of the pathology of the individual tumours have been described previously [20,28-30]. The RNA extraction process was based on standard Trizol methods, from which 100 μg per sample for the luminal pool and 50 μg total RNA for the malignant breast epithelial pool were used. All samples were stored at -80°C until being used in experiments. Informed consent was obtained to use this material for scientific research.

### MPSS, microarray and data processing

As described previously, both RNA pools were exploited by MPSS experiments at Lynx-Therapeutics, Inc (now Illumina, Hayward, CA) [31,32]. Briefly, following an RNA quality test on an Agilent 2100 BioAnalyzer (Agilent Technologies, Palo Alto, CA), cDNA libraries were generated from ~300 μg of DNase treated total RNA pools according to the "signature" Megaclone protocol [31,32]. The resulting libraries were amplified and yielded ~2 x 10^6 ^microbeads. The sequence adjacent to the poly(A)+ proximal *DpnII *site was determined by cycles of ligations to fluorescently tagged "decoding" oligonucleotides and cleavages by restriction enzymes. The abundance for each signature tag in the two pools was represented as transcripts per million (tpm), and sequence signatures seen in at least two independent runs and present at a frequency of at least 3 tpm in at least one sample were selected for further analysis.

Gene expression data in the normal luminal epithelial pool and the malignant breast epithelial pool was retrieved for the Affymetrix HG-U133 Plus 2.0 GeneChip^® ^(Affymetrix, Santa Clara, CA) platform from our previous study [20], as well as from the Almac Diagnostics Breast Cancer DSA [18]. Microarray experiments for the latter microarray platform were outsourced to ALMAC GROUP LTD, UK, and hybridisations, scanning and primary data acquisition were performed according to their manufacturer's protocols. Three technical replicates were obtained for each RNA pool. Briefly, 2 μg total RNA was reverse transcribed with a poly-(T) primer containing a T7 promoter, and the cDNA made double-stranded. An in vitro transcription was done to produce biotinylated cRNA, which was then hybridized to the GeneChips. The chips were washed and stained with streptavidin phycoerythrin using an Affymetrix FS-450 fluidics station, and data was collected with Affymetrix GeneChip Scanner 3000.

All data can be found on the ArrayExpress [33] website under E-TABM-657.

Pre-processing methods included normalisation and transformation, which were performed in the R-environment [34]. Since the Breast Cancer DSA is based on the Affymetrix GeneChip technology, normalisation and transformation of the raw data was performed using the robust multi-array analysis (RMA) [35] to obtain relative measurements for each probe sets. Secondly, analysis was performed on the Breast Cancer DSA with the Affymetrix Microarray Suite version 5 (MAS5) algorithms, which uses the probe-pair data to calculate the detection call. Based on a non-parametric Wilcoxon signed rank test of whether significantly more perfect matches show more hybridization signal than their corresponding mismatches to produce the detection call, MAS5 determines the absence or presence state for each probe sets [36].

### Matching of transcripts among microarray platforms and between MPSS and microarrays

A sequence-based approach was taken to match features across all different platforms. MPSS tags and microarray probes for the Breast Cancer DSA and Plus 2.0 were mapped to the Human Transcriptome (HTR) database developed by Iseli *et al*. [37-39] using the *tagger *software developed by Iseli. The mapping search was performed against both strands of the HTR database sequences to enable detection of both sense and antisense matches. Perfect homology was required across all 17 bases of the MPSS tags to produce a positive mapping. All 17 base tags began with the four base combination 'GATC' to ensure that all matches occurred immediately downstream of a *Dpn*II site. Microarray probes required perfect homology across all of their 25 bases to produce a positive match. A probe set (generally comprising 11 probes) required at least 5 probes to match a sequence in the same orientation to produce a positive mapping. HTR database cluster identifiers were retrieved for all positive matches. Furthermore, all microarray features that mapped to more than one HTR cluster were excluded from further analysis to avoid the one: many scenario where a microarray feature was linked to more than one HTR cluster, resulting in matches with multiple different microarray features of other platforms or multiple MPSS tags. However, several microarray features could map to the same HTR cluster, not only to avoid considerate reduction of the data, but also to have differentially regulated transcript isoforms represented.

### SAS pairs on Breast Cancer DSA

Full sequences used in the creation of the Breast Cancer DSA and the sequences of the 431 HTR clusters had low complexity regions and repeats masked using Paracel Filtering Package (Paracel inc. now Striking Development) [40]. The Exonerate software [41] was used to align the DSA sequences to the HTR clusters (score threshold 200 and ID threshold 90%) and custom PERL scripts used to process the output and search for overlap of sequences in sense-antisense orientation.

The database created by Galante *et al*. was obtained from the LICR Sense/Antisense portal [42] and formatted as a Paracel Blast database [40] Breast Cancer DSA probe selection regions for the SAS pairs where then blasted against the database. An alignment of 50% query length coverage and 90% identity was required to be considered a positive result. SAS pairs were considered to be novel when none of the probe selection regions representing them produced a positive alignment result.

### Strand-specific reverse transcription PCR (RT-PCR) analysis of cell lines and breast tumour samples

To determine fold changes in expression of a selected group of SAS pairs, quantitative real time PCR (qPCR) reactions were carried out as described by Sleeman *et al*. [43], using the ΔΔCT relative quantification method. The panel of breast cell lines comprised: 1098L, BT20, BT474, CAMA-1, GI101, HMT3552, Hs578T, MCF7, MDAMB231, MDAMB453, MDAMB468, PMC42, SKBR5, SKBR7, T47D and ZR75 and were a kind gift of Prof. Mike O'Hare. Ten infiltrating ductal carcinomas of histological grade 2 and 3 were retrieved from the Middlesex, UCL Hospital before 1996 and informed consent to use this material for scientific research was obtained. 100 ng of RNA was used to generate two independent cDNA syntheses for all samples using Omniscript Reverse Transcripion Kit (Qiagen, UK) as per manufacturer's guidelines. Primers for reverse transcription were designed to be gene and strand specific for both the sense and anti-sense strand (PrimerDesign Ltd, UK) for DCBLD2, MMP24 and KRT81. Analysis of qPCR data was performed using the immortalised luminal cell line (226L) as comparator for all the breast cell lines and solid tumours [see Additional file 6]. ACTB was used as endogenous control throughout all analysis. ΔΔCT relative quantification data are expressed as mean fold changes across samples together with 95% confidence intervals.

## Authors' contributions

AG designed the study, performed statistical analysis and drafted the manuscript. GRO participated in the design of the study, performed all sequence-level bioinformatics and drafted the manuscript. AT helped draft the manuscript. MJS and HK performed qPCR analysis. PSJ and AMN helped in the design of the study and edited the paper. All authors read and approved the final manuscript.

## Supplementary Material

Additional file 1 **Additional file **1. Mapping of Affymetrix probesets to HTR clusters with orientation and number of probes mapped. Click here for file

Additional file 2 **Additional file **2. Mapping of DSA probesets to HTR clusters with orientation and number of probes mapped. Click here for file

Additional file 3 **Additional file **3. Mapping of MPSS tags to HTR clusters with orientation. Click here for file

Additional file 4 **Additional file **4. Mapping of DSA probesets to 431 SAS containing HTR clusters expressed and validated by more than two technologies. Click here for file

Additional file 5 **Additional file **5. Mapping of DSA probesets to 163 unique SAS containing HTR clusters with representative public database identifiers. Click here for file

Additional file 6 **Additional file **6. Complete list of breast cancer cell lines used in the study and corresponding qPCR results Click here for file
